# Evaluating the implementation and effectiveness of antimicrobial stewardship interventions for surgical antimicrobial prophylaxis at a quaternary hospital in a lower-middle-income country: a secondary data analysis

**DOI:** 10.1017/ash.2026.10823

**Published:** 2026-09-08

**Authors:** Peter Kwamina McCarthy, Blessing Boakye Yiadom, Dennis Tsagli, Estherla Twene

**Affiliations:** 1 Washington University in St Louis: Bursky School of Public Health, USA; 2 Greater Accra Regional Hospital, Ghana

## Abstract

**Objective:**

To evaluate the implementation and effectiveness of handshake antimicrobial stewardship interventions for surgical antibiotic prophylaxis at a quaternary hospital in Ghana.

**Design:**

Retrospective cross-sectional secondary data analysis.

**Setting:**

Quaternary referral hospital in Accra, Ghana.

**Participants:**

Adult surgical encounters (n = 227) with complete antimicrobial stewardship documentation between June and September 2024.

**Methods:**

We analyzed antimicrobial stewardship audit records to assess recommendation acceptance, implementation, antibiotic selection concordance, and duration patterns. Outcomes were stratified by surgical service and procedure urgency. Descriptive statistics were used.

**Results:**

Of 227 encounters, 109 (48.0%) required stewardship recommendations, of which 78 (71.6%) were verbally accepted. However, only 39 (50.0% of accepted recommendations) were implemented. Implementation failures were primarily due to patient discharge (19.3%) and prescriber unavailability (16.5%). Duration optimization accounted for 76.1% of interventions. Overall, 43.2% of encounters exceeded the recommended prophylaxis duration (>24 hours). Emergency procedures had lower acceptance and higher missed opportunity rates compared to elective cases.

**Conclusions:**

Handshake stewardship demonstrated moderate acceptance but limited implementation. Execution failures were primarily associated with specific operational bottlenecks, including patient discharge and prescriber unavailability, which were exacerbated in emergency and high-volume surgical services. These findings suggest that in resource-limited settings, stewardship models must incorporate asynchronous communication and automated system safeguards (e.g., stop orders) to overcome workflow barriers.

## Introduction

### Background and rationale

Inappropriate antibiotic use in hospital settings contributes disproportionately to antimicrobial consumption and drives antimicrobial resistance (AMR),^1–3^ a burden that falls heavily on low- and middle-income countries (LMICs) where resource constraints compound stewardship challenges.^4^

In Ghana, AMR contributes significantly to surgical complication-related deaths.^5^ Antimicrobial stewardship programs (ASPs) are coordinated interventions designed to optimize antimicrobial use and minimize unintended consequences,^6^ with core activities including prospective audit and feedback.^7^ Antimicrobial “handshake stewardship,” a highly interactive model of prospective audit and feedback utilizing face-to-face rounding, has demonstrated high prescriber engagement and recommendation acceptance in high-income settings. However, most evidence originates from well-resourced environments, and there is limited empirical evidence describing its implementation fidelity or impact in LMIC contexts.

Pharmacists or infectious disease specialists conduct face-to-face rounds with prescribing teams to discuss antibiotic therapy in real time. This approach emphasizes collaborative dialogue rather than restrictive oversight, allowing prescribers to ask questions, review evidence, and adjust therapy during routine clinical workflows. Early evaluations of handshake stewardship have demonstrated high levels of prescriber engagement and recommendation acceptance.^8^ In a pediatric hospital program in the United States, Hurst et al reported acceptance rates approaching 80% following the implementation of daily handshake stewardship rounds. Subsequent studies have shown that this model can reduce broad-spectrum antibiotic use while maintaining clinical safety and high provider satisfaction. Long-term evaluations also suggest that handshake stewardship can remain sustainable over multiple years when integrated into routine clinical workflows. However, most evidence regarding the effectiveness of handshake stewardship originates from high-income settings with well-resourced stewardship teams, and there is limited empirical evidence describing its implementation or impact in low- and middle-income country contexts.

Surgical antibiotic prophylaxis represents a high-yield stewardship target: World Health Organization (WHO) and Infectious Diseases Society of America (IDSA) guidelines recommend single-dose or 24-h coverage for clean surgical procedures,^7,9^ yet prolonged durations (>24 h) remain pervasive globally, particularly in LMICs where prescribers often conflate prophylaxis with treatment.^4^

The University of Ghana Medical Centre (UGMC), a 1,000-bed quaternary referral hospital in Accra, implemented a pharmacist-led antimicrobial stewardship program in 2023, incorporating “handshake stewardship,” a rounding-based model where pharmacists engage prescribers face-to-face to optimize antibiotic regimens.^10,11^ While this approach has demonstrated effectiveness in high-resource settings,^10,11^ its implementation fidelity and impact in Ghanaian surgical contexts remain unquantified.

### Objectives

This secondary data analysis evaluates UGMC’s surgical prophylaxis stewardship activities between June and September 2024 across various surgical disciplines. The research report addresses four objectives:

1. Quantify acceptance and implementation rates of antimicrobial stewardship recommendations delivered through handshake stewardship.
2. Compare intended versus actual antibiotic selection and duration across surgical encounters.
3. Identify service-specific patterns in stewardship engagement and prescribing behavior.
4. Generate evidence-informed, context-specific recommendations for strengthening ASP processes at UGMC.

## Methods

### Study design and setting

This project employed a retrospective cross-sectional secondary data analysis design using antimicrobial stewardship audit records collected at UGMC between June 1 and September 30, 2024. Data were generated as part of routine pharmacist-led stewardship activities and reflect real-time reviews of surgical antibiotic prophylaxis prescribing across surgical services: Obstetrics and Gynecology, General Surgery, Orthopedic Surgery, ENT Surgery, Neurosurgery, Genitourinary Surgery, and other specialties (Cardiovascular/Thoracic, Plastic, Pediatric Surgery). The analysis focuses on implementation outcomes rather than clinical endpoints (eg, surgical site infections), consistent with program evaluation methodology for stewardship process assessment.^12^

### Participants

The source data set initially contained 245 surgical prophylaxis encounters. After excluding 18 records with incomplete documentation of critical variables (stewardship outcome, antibiotic stop date, or service type), the analytic sample comprised 227 de-identified encounters.

*Eligibility criteria*:

- Inclusion: Patients undergoing clean or clean-contaminated surgical procedures receiving systemic antibiotic prophylaxis with complete stewardship documentation
- Exclusion: Incomplete stewardship outcome documentation; contaminated/dirty procedures requiring therapeutic antibiotics; missing critical clinical variables

*Participant flow*:

- Potentially eligible records: 245
- Excluded due to incomplete documentation: 18
- Final analytic sample: 227 encounters (100% included in analysis)

**Figure 1.**
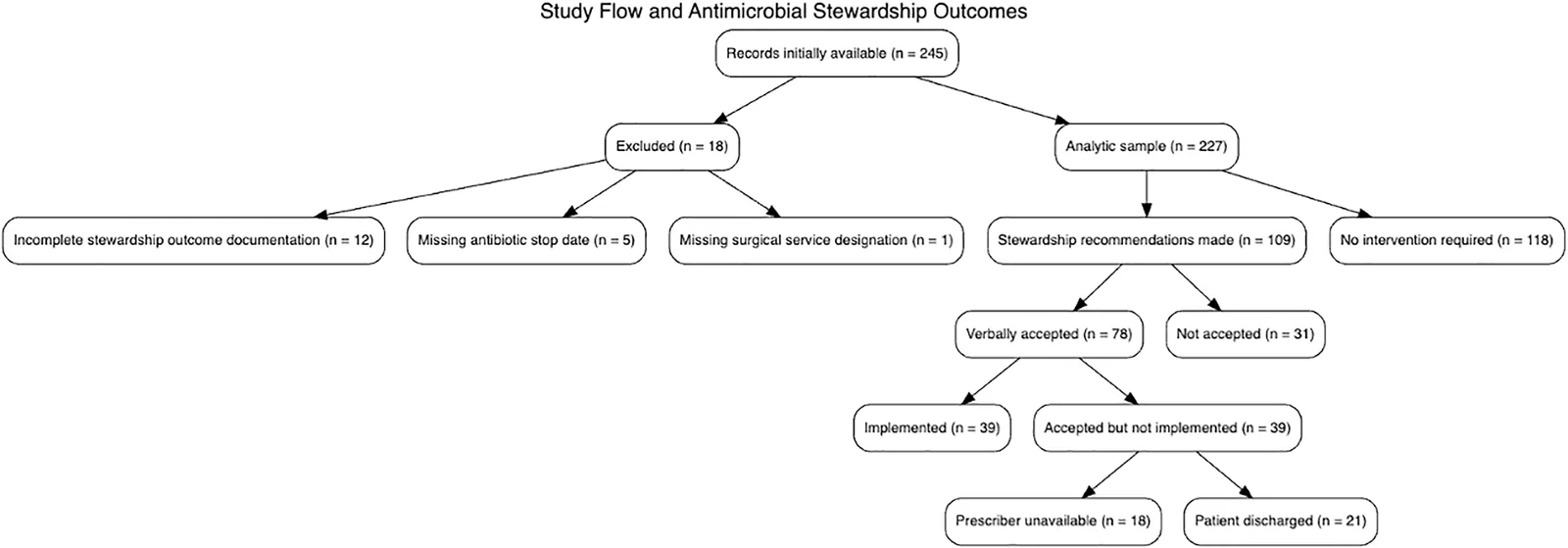
Figure 1 long description. Participant flow diagram.

No participants were lost to follow-up, as this constitutes a cross-sectional audit of completed encounters. Figure 1 presents the participant flow diagram.

### Variables and measures

Exposure: Antimicrobial stewardship intervention The primary exposure was receipt of an antimicrobial stewardship recommendation during surgical prophylaxis review. This was operationalized as a binary indicator (recommendation made: yes/no) derived from the *Stewardship outcome* field. Among encounters receiving a recommendation, intervention type was categorized as duration optimization, antibiotic choice adjustment, or a combined intervention (choice plus duration), based on entries in the *Intervention Type* field. Several stewardship implementation and prescribing outcomes were assessed as primary outcomes. Verbal acceptance of stewardship recommendations was defined as documentation of “Yes” in the *Recommendation verbally accepted field*. Stewardship implementation outcomes were categorized as implemented, accepted but not implemented due to patient discharge, accepted but not implemented due to prescriber unavailability, not accepted (missed opportunity), or no intervention required (appropriate prescribing), as recorded in the *Stewardship outcome* field.

Antibiotic selection concordance was defined as an exact match between the initially prescribed antibiotic and the antibiotic ultimately administered, using the *Initial antibiotic choice* and *Actual antibiotic choice* fields. Antibiotic duration was measured in days and calculated as the difference between the antibiotic stop date and start date plus one day. Intended duration and actual duration were analyzed both as continuous variables and categorized into clinically relevant duration groups for descriptive analyses. Clinical and procedural covariates included surgical service and procedure urgency (emergency vs elective). These variables were extracted directly from the corresponding audit fields and treated as categorical variables in the analysis. Barriers to stewardship implementation were identified among accepted recommendations that were not executed. These were categorized as prescriber unavailability or patient discharge, based on standardized text entries within the *Stewardship outcome* field.

### Statistical methods

All analyses were conducted using *R* version 4.3.2. Descriptive statistics summarized stewardship activities and prescribing patterns:

- Categorical variables: Frequencies and percentages
- Service-specific patterns: Stratified analysis by surgical service and procedure urgency
- Implementation gap decomposition:
  - *Acceptance gap*: Proportion of recommendations not verbally accepted
  - *Execution gap*: Proportion of accepted recommendations not implemented

Missing data (<1% for any variable) were handled through complete-case analysis, given minimal impact on estimates. No sensitivity analyses were required, given high data completeness. No formal sample size calculation was performed as this constitutes an analysis of an existing audit data set; the sample represents all eligible encounters during the 4-month audit period.

### Ethical considerations

This study was conducted as a secondary analysis of de-identified programmatic data and was determined to be exempt from human subjects review by the Washington University in St. Louis Institutional Review Board. Permission to use the data set for academic purposes was obtained from the University of Ghana Medical Centre Antimicrobial Stewardship team. Informed consent was not required due to the use of de-identified retrospective data.

## Results

### Participant characteristics

The analytic sample comprised 227 surgical prophylaxis encounters across seven services (Table 1). Obstetrics and Gynecology represented the highest-volume service (41.0%), followed by General Surgery (21.6%). Emergency procedures accounted for 23.8% of encounters. Cefuroxime was the predominant initial antibiotic choice (53.7%), followed by ceftriaxone (27.8%) and amoxicillin-clavulanate (17.6%).

**Table 1.** Characteristics of surgical prophylaxis encounters (n = 227) Table 1 long description.

Characteristic	n	%
**Surgical service**		
Obstetrics & gynecology	93	41.0
General surgery	49	21.6
Genitourinary surgery	26	11.5
Neurosurgery	20	8.8
Orthopedic surgery	19	8.4
ENT surgery	12	5.3
Other services^*^	8	3.5
**Procedure urgency**		
Emergency	54	23.8
Elective	173	76.2
**Antibiotic choice for prophylaxis**		
Cefuroxime	122	53.7
Ceftriaxone	63	27.8
Amoxicillin-clavulanate	40	17.6
Ciprofloxacin	2	.9

* Includes Cardiovascular/Thoracic, Plastic, Pediatric Surgery.

### Stewardship recommendation acceptance and implementation

Of the 227 encounters reviewed, 109 (48.0%) required antimicrobial stewardship recommendations, while 118 (52.0%) were deemed appropriate at baseline and required no intervention (Table 2).

**Table 2.** Stewardship recommendation outcomes (n = 227) Table 2 long description.

Outcome category	n	% of Total Encounters	% of Recommendations Made
Implemented (Accepted and implemented)	39	17.1%	35.8%
Accepted, not implemented (prescriber unavailable)	18	7.9%	16.5%
Accepted, not implemented (patient discharge)	21	9.3%	19.3%
Not accepted (missed opportunity)	31	13.7%	28.4%
No intervention required	118	52.0%	—
TOTAL	227	100.0%	100.0%

Among the 109 recommendations delivered:

- 78 (71.6%) were verbally accepted by the prescriber
- 39 (35.8% of recommendations; 17.2% of all encounters) were fully implemented
- 39 (35.8%) were accepted but not implemented, primarily due to:
  - Patient discharge prior to order execution: 21 cases (19.3% of recommendations)
  - Prescriber unavailable during handoffs or weekends: 18 cases (16.5% of recommendations)
- 31 (28.4%) were not accepted, representing missed stewardship opportunities

The implementation rate among accepted recommendations was 50.0% (39/78). These findings indicate a substantial execution gap: although most recommendations were verbally accepted, only half were ultimately implemented due to workflow-related barriers.

### Intended versus actual antibiotic selection and duration

Antibiotic selection: Concordance (initial choice = actual administered agent) occurred in 175 encounters (77.1%). Discordance (n = 52; 22.9%) primarily reflected stewardship-driven modifications (eg, ceftriaxone → cefuroxime conversions for orthopedic procedures; gentamicin addition for genitourinary surgery).

Duration optimization: Duration represented the predominant stewardship target. Distribution of intended durations is shown in Table 3 below.

**Table 3.** Distribution of intended preop prophylaxis duration (n = 227) Table 3 long description.

Intended duration category	n	%
≤24 hours (guideline-concordant)	129	56.8
2–4 days	27	11.9
5–7 days	60	26.4
≥8 days	11	4.8
TOTAL	227	100.0

It is important to note that 43.2% of encounters (98/227) had intended durations of preop prophylaxis exceeding 24 hours, contrary to WHO/IDSA guidelines.^7^

Across all recommendations, duration optimization accounted for 76.1% of interventions (83/109), followed by combined duration and antibiotic choice interventions (16/109, 14.7%) and antibiotic choice adjustments alone (10/109, 9.2%).

### Service-specific patterns in stewardship engagement

Substantial variability in stewardship engagement was observed across surgical services (Table 4). Acceptance rates ranged from 62.1% to 100.0%, while implementation rates (as a proportion of the recommendations made) ranged from 0% to 83.3%.

**Table 4.** Service-specific stewardship metrics (n = 227) Table 4 long description.

Surgical service	Total encounters	Recommendations made, n (%)	Accepted, n	Implemented, n	Not accepted, n	Acceptance rate (%)	Implementation rate^†^(%)	Missed opportunity Rate (%)
Obstetrics & gynecology	93	29 (31.2)	18	13	11	62.1	44.8	37.9
General surgery	49	32 (65.3)	24	12	8	75.0	37.5	25.0
Genitourinary surgery	26	20 (76.9)	15	0	5	75.0	.0	25.0
Neurosurgery	20	9 (45.0)	6	3	3	66.7	33.3	33.3
Orthopedic surgery	19	6 (31.6)	6	5	0	100.0	83.3	.0
ENT surgery	12	10 (83.3)	7	6	3	70.0	60.0	30.0
Other services^*^	8	3 (37.5)	2	0	1	66.7	.0	33.3
TOTAL	227	109 (48.0)	78	39	31	71.6	35.8	28.4

* Includes Cardiovascular/Thoracic, Plastic, Pediatric Surgery.

† Implementation Rate = implemented ÷ recommendations made.

Lower-volume services with standardized prophylaxis practices (eg, Orthopedic, ENT) tended toward higher acceptance and implementation, whereas high-volume Obstetrics and Gynecology showed moderate acceptance (62.1%) but limited implementation (44.8%). Emergency procedures were accepted less often than elective procedures, with a correspondingly higher missed-opportunity rate. These service- and urgency-level patterns are descriptive and unadjusted and are not interpreted as independent effects.

## Discussion

### Principal findings

This evaluation of a pharmacist-led handshake stewardship program at a quaternary hospital in Ghana identified two gaps limiting stewardship effectiveness: an acceptance gap (28.4% of recommendations declined) and an execution gap (50.0% of accepted recommendations not implemented). While roughly three-quarters of recommendations were verbally accepted, only half were ultimately implemented, suggesting that prescriber agreement alone may be insufficient to ensure prescribing change without better workflow integration. Duration optimization was the primary stewardship need, accounting for 76.1% of interventions; antibiotic selection was generally appropriate at baseline (77.1% concordance), whereas prolonged prophylaxis (>24 h in 43.2% of cases) remained the dominant driver of potentially inappropriate antibiotic exposure.^14^

### Comparison with existing literature

Our 71.6% verbal acceptance aligns with Hurst et al., whose evaluation of pediatric handshake stewardship reported 78% acceptance, confirming the model’s effectiveness for engagement.^10,13^ However, our 50% implementation rate falls well below the 86% reported by MacBrayne et al in a US sustainability study.^11^ This discrepancy is consistent with resource constraints in LMIC settings, where 24/7 pharmacist coverage is often unavailable, so recommendations agreed upon verbally are frequently lost when patients are discharged before the change is enacted or when the prescriber is later unavailable to execute it.^4^ The barriers we observed were therefore less about clinical disagreement than about the logistics of converting a verbal, face-to-face agreement into a documented order under limited staffing. Our 43.2% rate of prophylaxis exceeding 24 hours is also notably higher than the 22% reported by Elrefaei et al in a US quaternary hospital, consistent with Otieno et al.’s finding that prolonged duration is a persistent challenge in sub-Saharan African programs.^5,15^

### Service- and procedure-level drivers

Stewardship outcomes also varied with service and procedural context (Table 4). Recommendations were less consistently accepted and implemented in emergency procedures and in high-volume Obstetrics and Gynecology (62.1% acceptance, 44.8% implementation) than in lower-volume services with more standardized prophylaxis practices, such as Orthopedic Surgery (100.0% acceptance, 83.3% implementation). This descriptive pattern is consistent with time pressure, heightened risk perception, and workflow disruptions in emergency and high-throughput settings, which may limit adherence to guidelines even when stewardship input is available.^2^ Comparable service-level gaps have been reported in resource-limited settings relative to higher-income contexts^17^ and appear to reflect specialty-specific workflow and prophylaxis norms rather than resistance to stewardship principles.^12^ These observations suggest that the handshake model is most fragile precisely where it is most needed: in high-acuity, high-volume environments whose pace and unpredictability disrupt the real-time, face-to-face interaction the model depends on.

### Implications for antimicrobial stewardship programs

These findings point to specific, feasible adaptations rather than generic calls for additional resources. Because excessive duration, not agent choice, was the dominant problem, embedding automatic stop orders for standard clean procedures within surgical order sets could prevent prolonged prophylaxis without requiring pharmacist input on every case.^18^ Because discharge timing was a leading cause of execution failure, routine predischarge pharmacy review of surgical prophylaxis would allow recommendations to be enacted before the patient leaves. And because prescriber unavailability repeatedly blocked implementation, empowering pharmacists to execute preagreed, protocolized duration limits through electronic stop orders rather than relying on repeated face-to-face verbal orders would decouple implementation from the physical presence of the prescriber.^19,20^

### Strengths and limitations

Strengths: This study provides real-world LMIC data with granular documentation of the specific barriers to stewardship implementation, complete capture of stewardship interactions during the audit period, and a focus on implementation outcomes that are often overlooked in favor of clinical endpoints alone.

Limitations: Several limitations should be considered. First, the single-center retrospective design limits generalizability. Second, we excluded 18 encounters (7.3%) because of incomplete documentation, which may introduce selection bias; incomplete documentation could occur disproportionately during high-volume periods, emergency cases, or understaffed shifts, meaning our sample may underrepresent the most chaotic clinical scenarios in which stewardship failures are most likely. For the same reason, our implementation gap (50.0%) may partly reflect underreporting of implemented changes rather than true implementation. Third, as a descriptive analysis with a limited sample size, we did not perform multivariable adjustment for potential confounders; differences between emergency and elective procedures should therefore be interpreted as associative rather than causal. Fourth, the data set was limited to surgical prophylaxis and did not capture medical antibiotic use. Finally, this study evaluated process and implementation outcomes rather than clinical endpoints such as surgical site infections or readmissions. Although clinical outcomes are the ultimate measure of stewardship success, prolonged prophylaxis beyond 24 hours is a recognized marker of potentially avoidable antibiotic exposure,^21,22^ and evaluating implementation fidelity is a necessary first step in program evaluation, particularly in settings where robust clinical outcome surveillance may be limited.

### Generalizability

Findings are generalizable to similar quaternary hospitals in LMIC settings implementing pharmacist-led handshake stewardship models. The predominance of duration-related prescribing gaps and workflow barriers to implementation likely reflects systemic challenges common across resource-limited surgical settings. However, service-specific patterns may vary based on local prescribing cultures and procedural case mixes. Our findings regarding the impact of emergency procedures on stewardship outcomes are particularly relevant for LMIC settings where emergency surgical volumes often exceed those in high-income countries.^4,21^

In conclusion, the antimicrobial stewardship recommendations delivered through handshake stewardship are frequently accepted but inconsistently implemented. Excessive duration of surgical prophylaxis, not antibiotic choice, represents the principal prescribing gap. Emergency procedures and workflow disruptions substantially undermine stewardship effectiveness. Future stewardship efforts in similar settings should prioritize embedded system solutions, such as automatic stop orders and workflow-aligned interventions, to translate accepted recommendations into sustained prescribing change. These findings support the growing evidence that effective stewardship in LMIC settings requires context-specific adaptation rather than direct implementation of high-income country models.

## Data Availability

The data that support the findings of this study are available from the corresponding author upon reasonable request.

## Figure 1. Long description

A flowchart illustrating the study flow and antimicrobial stewardship outcomes. The flowchart begins with 245 records initially available. 18 records are excluded due to incomplete stewardship outcome documentation (12), missing antibiotic stop date (5), and missing surgical service designation (1). This leaves an analytic sample of 227 records. The analytic sample is divided into two main branches: stewardship recommendations made (109) and no intervention required (118). For the 109 records where stewardship recommendations were made, 78 were verbally accepted and 31 were not accepted. Of the 78 verbally accepted recommendations, 39 were implemented and 39 were accepted but not implemented. The 39 implemented recommendations further split into prescriber unavailable (18) and patient discharged (21).

Navigate back to Figure 1..

## Table 1. Long description

A table with 11 rows and 3 columns detailing characteristics of surgical prophylaxis encounters. The columns are labeled Characteristic, n, and %. The table includes data on surgical service, procedure urgency, and antibiotic choice for prophylaxis. Surgical services listed are Obstetrics & Gynecology, General Surgery, Genitourinary Surgery, Neurosurgery, Orthopedic Surgery, ENT Surgery, and Other Services. Procedure urgency is divided into Emergency and Elective. Antibiotic choices include Cefuroxime, Ceftriaxone, Amoxicillin-clavulanate, and Ciprofloxacin. Notable data points include Obstetrics & Gynecology having the highest number of encounters (93, 41.0%), and Cefuroxime being the most commonly chosen antibiotic (122, 53.7%).

Navigate back to Table 1..

## Table 2. Long description

A table with five columns and six rows, including a header row. The columns are labeled Outcome category, n, % of Total Encounters, and % of Recommendations Made. The rows detail different outcome categories and their respective values. Row 1: Outcome category, Implemented (Accepted and implemented); n, 39; % of Total Encounters, 17.1 percent; % of Recommendations Made, 35.8 percent. Row 2: Outcome category, Accepted, not implemented (prescriber unavailable); n, 18; % of Total Encounters, 7.9 percent; % of Recommendations Made, 16.5 percent. Row 3: Outcome category, Accepted, not implemented (patient discharge); n, 21; % of Total Encounters, 9.3 percent; % of Recommendations Made, 19.3 percent. Row 4: Outcome category, Not accepted (missed opportunity); n, 31; % of Total Encounters, 13.7 percent; % of Recommendations Made, 28.4 percent. Row 5: Outcome category, No intervention required; n, 118; % of Total Encounters, 52.0 percent; % of Recommendations Made, not applicable. Row 6: Outcome category, TOTAL; n, 227; % of Total Encounters, 100.0 percent; % of Recommendations Made, 100.0 percent.

Navigate back to Table 2..

## Table 3. Long description

A table showing the distribution of intended preop prophylaxis duration across different categories. The table has four rows and three columns. The columns are labeled Intended duration category, n, and %. The row labels and values are as follows: Row 1: <= 24 hours (guideline-concordant), 129, 56.8 percent. Row 2: 2-4 days, 27, 11.9 percent. Row 3: 5-7 days, 60, 26.4 percent. Row 4: >=8 days, 11, 4.8 percent. The total row is labeled TOTAL, with values 227 and 100.0 percent.

Navigate back to Table 3..

## Table 4. Long description

The table presents stewardship metrics for various surgical services, detailing total encounters, recommendations made, accepted recommendations, implemented recommendations, not accepted recommendations, acceptance rate, implementation rate, and missed opportunity rate. The table has 8 rows and 9 columns. The columns are labeled as follows: Surgical service, Total encounters, Recommendations made, n (%), Accepted, n, Implemented, n, Not accepted, n, Acceptance rate (%), Implementation rate (%), and Missed opportunity Rate (%). The rows are labeled with different surgical services: Obstetrics & gynecology, General surgery, Genitourinary surgery, Neurosurgery, Orthopedic surgery, ENT surgery, Other services, and TOTAL. Row 1: Obstetrics & gynecology, 93, 29 (31.2), 18, 13, 11, 62.1, 44.8, 37.9. Row 2: General surgery, 49, 32 (65.3), 24, 12, 8, 75.0, 37.5, 25.0. Row 3: Genitourinary surgery, 26, 20 (76.9), 15, 0, 5, 75.0, 0.0, 25.0. Row 4: Neurosurgery, 20, 9 (45.0), 6, 3, 3, 66.7, 33.3, 33.3. Row 5: Orthopedic surgery, 19, 6 (31.6), 6, 5, 0, 100.0, 83.3, 0.0. Row 6: ENT surgery, 12, 10 (83.3), 7, 6, 3, 70.0, 60.0, 30.0. Row 7: Other services, 8, 3 (37.5), 2, 0, 1, 66.7, 0.0, 33.3. Row 8: TOTAL, 227, 109 (48.0), 78, 39, 31, 71.6, 35.8, 28.4.

Navigate back to Table 4..
